# Contribution of Human Immunodeficiency Virus Type 1 Minority Variants to Reduced Drug Susceptibility in Patients on an Integrase Strand Transfer Inhibitor-Based Therapy

**DOI:** 10.1371/journal.pone.0104512

**Published:** 2014-08-11

**Authors:** Richard M. Gibson, Jan Weber, Dane Winner, Michael D. Miller, Miguel E. Quiñones-Mateu

**Affiliations:** 1 University Hospital Translational Laboratory, University Hospitals Case Medical Center, Cleveland, Ohio, United States of America; 2 Institute of Organic Chemistry and Biochemistry, Prague, Czech Republic; 3 Gilead Sciences, Inc., Foster City, California, United States of America; 4 Department of Pathology, Case Western Reserve University, Cleveland, Ohio, United States of America; University of Pittsburgh, United States of America

## Abstract

The role of HIV-1 minority variants on transmission, pathogenesis, and virologic failure to antiretroviral regimens has been explored; however, most studies of low-level HIV-1 drug-resistant variants have focused in single target regions. Here we used a novel HIV-1 genotypic assay based on deep sequencing, DEEPGEN (Gibson et al 2014 Antimicrob Agents Chemother 58∶2167) to simultaneously analyze the presence of minority variants carrying mutations associated with reduced susceptibility to protease (PR), reverse transcriptase (RT), and integrase strand transfer integrase inhibitors (INSTIs), as well as HIV-1 coreceptor tropism. *gag*-p2/NCp7/p1/p6/*pol*-PR/RT/INT and *env*/C2V3 PCR products were obtained from twelve heavily treatment-experienced patients experiencing virologic failure while participating in a 48-week dose-ranging study of elvitegravir (GS-US-183-0105). Deep sequencing results were compared with (i) virological response to treatment, (ii) genotyping based on population sequencing, (iii) phenotyping data using PhenoSense and VIRALARTS, and (iv) HIV-1 coreceptor tropism based on the phenotypic test VERITROP. Most patients failed the antiretroviral regimen with numerous pre-existing mutations in the PR and RT, and additionally newly acquired INSTI-resistance mutations as determined by population sequencing (mean 9.4, 5.3, and 1.4 PI- RTI-, and INSTI-resistance mutations, respectively). Interestingly, since DEEPGEN allows the accurate detection of amino acid substitutions at frequencies as low as 1% of the population, a series of additional drug resistance mutations were detected by deep sequencing (mean 2.5, 1.5, and 0.9, respectively). The presence of these low-abundance HIV-1 variants was associated with drug susceptibility, replicative fitness, and coreceptor tropism determined using sensitive phenotypic assays, enhancing the overall burden of resistance to all four antiretroviral drug classes. Further longitudinal studies based on deep sequencing tests will help to clarify (i) the potential impact of minority HIV-1 drug resistant variants in response to antiretroviral therapy and (ii) the importance of the detection of HIV minority variants in the clinical practice.

## Introduction

Antiretroviral therapy based on the combination of several anti-HIV-1 drugs is the gold standard of care for HIV-1 infected individuals in most developed countries and has been credited with considerable reductions in morbidity and mortality [1], [2]. Commonly prescribed antiretroviral regimens include two nucleoside reverse transcriptase inhibitors (NRTIs) in combination with a nonnucleoside reverse transcriptase inhibitor (NNRTI), a protease inhibitor (PI), or an integrase strand transfer inhibitor (INSTI) [3], [4]. INSTI is the most recent class of antiretroviral drugs approved by the U.S. Food and Drug Administration (FDA) for the treatment of HIV-1 infection. Raltegravir (RAL, MK-0518, Isentress, Merck & Co., Inc.) was the first INSTI approved in 2007 [5]. Elvitegravir (EVG, JTK-303/GS-9137, Gilead Sciences)[6] was approved in 2012 in a fixed dose combination with a pharmacokinetic enhancer (cobicistat) and two nucleos(t)ide analog RT inhibitors (emtricitabine and tenofovir) for the treatment of antiretroviral-naïve HIV-infected individuals (QUAD, Stribild, Gilead Sciences) [7]. Dolutegravir (DTG, S/GSK1349572, Tivicay, GlaxoSmithKline), a second-generation INSTI [8], is the latest INSTI available to treat HIV infection [9], [10]. Unfortunately, despite the success of more potent and safe antiretroviral drugs with simpler regimens, current therapies have not been able to eradicate latent reservoirs of HIV-1 [11], [12]. Moreover, the emergence [2], [13] and potential transmission [14] of HIV-1 drug-resistant variants continues to be a cause of virologic failure in patients treated with combinations of antiretroviral drugs [2], [15].

HIV-1, like other RNA viruses, has a high mutation rate (approximately 10^−4^ to 10^−5^ mutations per nucleotide and cycle of replication [16]), which coupled with selection and rapid turnover [17] results in the generation of swarms of mutants known as viral quasispecies (HIV-1 variants) [18], [19], [20]. HIV-1 is constantly evolving and adapting, exploring all potential combinations of mutations that could increase the capacity of the virus to replicate in any given environment (replicative fitness), including the generation and potential selection of strains carrying drug resistance mutations [21], [22]. Therefore, all drug-resistant HIV-1 strains arise as the consequence of the introduction of one -or more- incorrect nucleotide during virus replication in the absence of proofreading mechanisms [23]. These drug-resistant variants will initially be present as minority members of the virus population, which could eventually be selected and outcompete other variants depending of their ability to replicate under drug pressure [21], [22], [24]. Current genotypic HIV-1 drug resistance assays based on population (Sanger) sequencing are able to detect these minority variants when their frequency reaches approximately 20% of the virus population [25], [26], [27], [28], [29]; however, early detection of drug resistant HIV-1 minority variants has been associated with the ability to predict clinical outcome [30], [31], [32], [33], [34], [35]. Thus, ultrasensitive HIV-1 genotyping assays based on allele-specific polymerase chain reaction [36], [37], oligonucleotide ligation [38], [39], or deep sequencing [40], [41], [42], [43], which are capable of detecting drug-resistant minority HIV-1 variants below the 20% level, may help to clarify the actual clinical relevance of these minority members of the viral population [30], [39], [44], [45], [46].

In this study we used a novel HIV-1 genotyping assay based on deep sequencing (DEEPGEN)[43] to detect and quantify low-level drug-resistant HIV-1 variants in twelve patients experiencing virologic failure while participating in a 48-week dose-ranging study of elvitegravir (GS-US-183-0105). Deep sequencing-based HIV-1 genotypes were then compared with (i) mutation profiles obtained by standard population sequencing, (ii) drug susceptibility using HIV-1 phenotypic assays, (iii) HIV-1 replicative fitness, and (iv) virological response to antiretroviral treatment.

## Materials and Methods

### Clinical samples

Plasma samples were obtained from twelve patients experiencing virologic failure while participating in a 48-week dose-ranging study of elvitegravir (EVG), Study GS-US-183-0105 [47](Table 1). The Western IRB (Olympia, WA) and Chesapeake IRB (Columbia, MD) approved Gilead Study 183-0105, and written informed consent was obtained from all study subjects as previously described [47], [48].

**Table 1:** Virological parameters of 12 HIV-infected individuals participating in the GS-US-183-0105 study of elvitegravir.

		Major Mutations in INT^a^								EC_50_ FC INSTI		EC_50_ FC PI									EC_50_ FC NNRTI				EC_50_ FC NRTI						
Patient	HIV-1 RNA^b^	E92	N155	Q148	PI (1)	PI (2)	#TAMs	#NAMs	NNRTI (1)	EVG	RAL	APV	TPV	RTV	IDV	NFV	DRV	SQV	LPV	ATV	EFV	NVP	ETR	DLV	AZT	ABC	3TC	FTC	d4T	TFV	ddI
08–175	4.75				5	5	5	7	2	17	0.9	17	0.8	MAX	7.6	34	6.0	67	32	44	MAX	133	6.5	210	4.2	12	MAX	MAX	4.0	1.5	2.2
08–172	4.77	E92E/Q			6	4	5	7	3	3.0	0.7	37	3.2	MAX	19	369	7.4	MAX	MAX	239	MAX	MAX	22	6.5	88	12	211	MAX	23	9.1	5.5
08–198	5.58	E92E/Q			0	3	1	2	0	1.2	1.4	0.9	0.7	3.4	0.6	2.1	0.8	2.7	2.9	2.0	2.8	3.1	0.7	3.1	6.2	1.8	5.1	15	2.9	2.2	0.7
08–183	5.24	E92Q			3	4	5	6	0	7.8	2.3	3.2	1.8	7.8	3.3	54	1.6	30	21	45	8.6	5.8	0.9	1.0	9.3	7.5	267	MAX	3.4	5.5	4.6
08–180	5.43	E92Q			8	6	4	6	0	37	1.9	MAX	1.8	MAX	4.5	MAX	MAX	66	MAX	MAX	6.5	4.8	0.7	1.5	40	4.9	MAX	MAX	5.0	5.5	3.8
08–177	5.03		N155N/H		7	5	4	6	2	29	2.2	45	1.3	MAX	16	126	19	19	MAX	65	44	MAX	475	29	19	5.7	166	317	14	7.1	2.9
08–194	5.66		N155H		3	5	2	4	1	49	2.6	35	2.5	129	4.2	25	2.7	8.8	15	7.8	9.2	5.8	1.5	0.5	5.6	5.5	MAX	MAX	2.9	1.4	3.4
08–201	5.75		N155H		3	2	0	1	0	128	3.2	2.9	0.9	7.6	2.0	14	1.0	0.9	4.9	1.0	2.6	1.6	1.3	0.5	1.0	3.4	MAX	MAX	1.6	0.8	1.4
08–202	5.33	E92E/Q	N155N/H		4	2	2	2	1	4.2	1.2	0.8	0.5	5.1	1.5	13	0.7	1.8	0.2	1.3	MAX	MAX	0.3	19	13	2.0	0.6	0.9	3.6	4.9	1.1
08–230	4.55	E92Q	N155H		4	10	5	7	1	492	18	18	2.7	MAX	MAX	MAX	3.3	MAX	MAX	MAX	65	30	0.5	47	18	9.0	MAX	MAX	3.8	4.4	2.4
08–189	5.76			Q148R	2	2	3	4	0	35	2.3	5.8	1.4	13	17	38	1.2	1.0	7.5	13	1.6	4.5	0.5	0.6	18	7.6	244	161	19	10	3.2
08–182	5.50			Q148R	3	0	0	3	2	179	5.4	66	0.6	8.8	1.1	7.7	7.8	3.8	7.8	9.2	MAX	MAX	293	133	1.9	13	MAX	MAX	7.2	2.9	8.4

a Major mutations associated with resistance to INSTI as described [71], [86].

b Plasma viral load (log10 copies/ml). INSTI-R, mutations associated with resistance to INSTI; PI (1), number of primary mutations associated with resistance to PI; PI (2), number of secondary mutations associated with resistance to PI; #TAMs, number of thymidine analogue-associated mutations; #NAMs, number of nucleoside analogue-associated mutations; NNRTI (1), number of primary mutations associated with resistance to NNRTI. EC_50_ FC, based on VIRALARTS [49] three independent EC_50_ replicates for each drug were used to calculate the fold changes (FC) of the query viruses relative to the HIV-1_NL4-3_ control and the mean EC_50_ FC is indicated. MAX, complete virus inhibition was not achieved using the maximum drug concentration, i.e., virus was completely resistant to the respective antiretroviral drug. Virus 08-175 contained the INSTI-resistance mutations T66A and S147G.

### Cells

MT-4 (Dr. D. Richman), U87.CD4.CCR5, and U87.CD4.CXCR4 cells (Drs. H. Kui and D. Littman) were obtained from the AIDS Research and Reference Reagent Program, Division of AIDS, NIAID, NIH and the HEK293T cells from Stanford University (Stanford, CA). MT-4 cells were maintained in RPMI 1640/2 mM L-glutamine medium (Cellgro; Mediatech, Herndon, VA) supplemented with 10% fetal bovine serum (FBS; Cellgro), 10 mM N-2-hydroxyethylpiperazine-N-2-ethanesulfonic acid buffer (HEPES; Sigma-Aldrich, St. Louis, MO), 100 U of penicillin/ml, and 100 µg of streptomycin/ml (Gibco; Invitrogen, Carlsbad, CA). U87.CD4.CCR5 and U87.CD4.CXCR4 cells were maintained in DMEM medium with L-glutamine (Cellgro; Mediatech) supplemented with 15% fetal bovine serum, 100 U of penicillin/mL, 100 µg of streptomycin/mL, 1 µg/ml of puromycin, and 300 µg of G418 (all reagents from Mediatech). HEK293T were maintained in DMEM medium/L-glutamine (Gibco), 10% FBS (Cellgro), and penicillin/streptomycin (Gibco).

### Antiretroviral drugs

The antiretroviral drugs used in this study were obtained from the following sources: zidovudine, AZT; didanosine, ddI; stavudine, d4T; lamivudine, 3TC; abacavir, ABC; tenofovir, TDF; emtricitabine, FTC; nevirapine, NVP; delavirdine, DLV; efavirenz, EFV; etravirine, ETR; saquinavir, SQV; ritonavir, RTV; indinavir, IDV; nelfinavir, NFV; amprenavir, APV; lopinavir, LPV; atazanavir, ATV; tipranavir, TPV; and darunavir, DRV (ENZO Life Sciences International, Inc., Plymouth Meeting, PA, formerly BioMol International, LP); raltegravir, RAL and elvitegravir, EVG (Gilead Sciences, Inc., Foster City, CA).

### Reverse transcription (RT)-PCR amplification of *gag*-p2/NCp7/p1/p6/*pol*-PR/RT/IN- and *env*-C2V3-coding regions

Plasma viral RNA was purified from pelleted virus particles by centrifuging one milliliter of plasma at 20,000 g×60 min at 4°C, removing 860 µl of cell-free supernatant and resuspending the pellet in the remaining 140 µl, to finally extract viral RNA using QIAamp Viral RNA Mini kit (Qiagen; Valencia, CA). Viral RNA was reverse-transcribed using AccuScript High Fidelity Reverse Transcriptase (Stratagene Agilent; Santa Clara, CA) and the corresponding antisense external primers in 20 µl reaction mixture containing 1 mM dNTPs, 10 mM DTT and 10 units of RNAse inhibitor. The HIV-1 genomic region encoding the Gag proteins p2, p7, p1 and p6, and the protease, reverse transcriptase, and integrase enzymes was amplified as two overlapping fragments (1,657 nt and 2,002 nt corresponding to the p2–5′half RT and 3′half RT-INT, respectively) using a series of external and nested primers with defined cycling conditions [49]. External PCR reactions were carried out in a 50-µl mixture containing 0.2 mM dNTPs, 3 mM MgCl_2_ and 2.5 units of Pfu Turbo DNA Polymerase (Stratagene). Nested PCR reactions were carried out in 50-µl mixture containing 0.2 mM dNTPs, 0.3 units of Pfu Turbo DNA Polymerase and 1.9 units of Taq Polymerase (Denville Scientific; Metuchen, NJ). A fragment corresponding to the C2V3 region (480 nt) of the surface glycoprotein (gp120) in the envelope gene was amplified using a series of external and nested primers with defined cycling conditions as previously described [50].

### Construction of *gag*-p2/NCp7/p1/p6/*pol*-PR/RT/IN-recombinant viruses

Infectious recombinant viruses were constructed from each clinical samples in a HIV-1_NL4-3_ backbone using a novel yeast-based cloning technology as described [49]. Briefly, PCR products spanning the 3′ end of *gag* (p2/p7/p1/p6) and the entire *pol* gene (PR/RT/IN; p2-INT) were introduced via yeast homologous recombination into pRECnfl-TRPΔp2-INT/URA3 or pRECnfl-TRPΔINT/URA3 vectors, respectively, containing a near-full length HIV-1 genome with the yeast uracil biosynthesis (URA3) gene replacing the respective p2-INT HIV-1 coding sequences. Following yeast transformation, vector DNA was purified from the entire number of yeast colonies (typically 200 to >1,000 individual colonies) and used to transform Electrocomp TOP10 bacteria (Invitrogen). Plasmid DNA from the entire bacteria preparation – to guarantee the continuity of the viral population that may have existed in vivo – was purified from 10 ml of bacteria culture (QIAprep Spin Miniprep Kit, Qiagen) and used to introduce the patient-derived HIV-1 sequences into a pNL4-3-hRluc vector expressing the human Renilla luciferase gene (hRluc) [51] as described [49]. Four micrograms of the resulting plasmid were transfected into HEK293T cells using GenDrill (BamaGen Bioscience; Gaithersburg, MD). Cell culture supernatant was harvested 48 hours post-transfection, clarified by centrifugation at 700×g, filtered through a 0.45 µm steriflip filter (Millipore; Billerica, MA), aliquoted, and stored at −80°C until further use. Tissue culture dose for 50% infectivity (TCID_50_) was determined in triplicate for each serially diluted virus using the Reed and Muench method [52] and viral titers expressed as infectious units per milliliter (IU/ml).

### Drug susceptibility determination using VIRALARTS

Drug susceptibility of twelve p2-INT-recombinant viruses was measured by determining the extent to which antiretroviral drugs inhibited viral replication in MT-4 cells as described [49]. Briefly, serial dilutions spanning empirically determined ranges of each drug were added in triplicate in 96-well plates in RPMI medium with L-glutamine (Cellgro; Mediatech) supplemented with 10% fetal bovine serum, 100 U of penicillin/mL, 100 µg of streptomycin/mL, (Mediatech) and 10 mM HEPES (Sigma-Aldrich). MT-4 cells were infected with either the reference virus (HIV-1_NL4-3-hRluc_) [51] or the corresponding query virus (HIV-1_p2-INT-hRluc_) expressing human Renilla luciferase at a multiplicity of infection (MOI) of 0.005 IU/cell for one hour at 37°C, 5% CO_2_. HIV-infected MT-4 cells were then resuspended in RPMI medium and 30,000 cells were added to each well containing pre-plated antiretroviral drugs. Virus replication was quantified 72 hours post-infection by measuring renilla luciferase activity (relative light units, RLU) using the *Renilla* Luciferase Assay System (Promega, Madison, WI) in a multiwell plate reader (Victor V multilabel reader, PerkinElmer, Waltham, MA). Drug concentrations required to inhibit virus replication by 50% (EC_50_) were calculated by (i) plotting the percent inhibition of luciferase activity versus log_10_ drug concentration and (ii) fitting the inhibition curves to the data using nonlinear regression analysis (GraphPad Prism v.6.0b, GraphPad Software, La Jolla, CA). Fold change (FC) resistance values were calculated by dividing the mean EC_50_ of the query virus (HIV-1_p2-INT-hRluc_) by the mean EC_50_ of the internal control (HIV-1_NL4-3-hRluc_) in each assay.

### HIV-1 coreceptor tropism determination using VERITROP

The ability of the virus to use the chemokine receptors CCR5 and/or CXCR4 as coreceptors to enter the host cell was quantified using a novel assay based on a modified version of the α-complementation assay for HIV-envelope glycoprotein-mediated fusion [53] as previously described [54]. Briefly, patient-derived PCR products spanning the gp120/gp41-coding region of HIV-1 were introduced via yeast homologous recombination into the pRECnfl-LEU-ΔEnv(gp120-tatex2)/URA3 vector containing a near-full length HIV-1 genome with the yeast uracil biosynthesis (URA3) gene replacing the gp120/gp41 HIV-1 coding sequence. Following yeast and bacteria transformation, 2 µg of the HIV-expression vector and 2 µg of a vector expressing the α fragment of the β-galactosidase gene (pCMVα) were co-transfected into 7×10^5^ HEK293T (donor) cells using Lipofectamine 2000 (Invitrogen). The target cells (U87.CD4.CCR5 or U87.CD4.CXCR4) were transfected with 4 µg of a vector expressing the omega fragment (pCMVϖ) of the β-galactosidase gene. Forty-eight hours post-transfection the donor and target cells were washed, removed from the cell-culture plates, counted and re-suspended in DMEM at a concentration of 2×10^6^ cells per milliliter. Fifty microliters (1×10^5^) of donor and target cells were mixed and added together into 96-well plate and incubated for 4 hours at 37°C in 5% CO_2_. Cell-to-cell fusion events were quantified by measuring luminescence related to β-galactosidase activity (relative light units, RLU) using Galacto-star system (Applied Biosystems, Bedford, MA) in a multi-well plate reader (Victor V multilabel reader, PerkinElmer, Waltham, MA). Controls were run in each test, including mock cell and transfections with plasmid DNA mixtures containing (i) 100%+0%, (ii) 1%+99%, (iii) 0.3%+99.7%, and (iv) 0%+100% of vectors expressing the *env* gene from the ×4 HIV-1_NL4-3_ or the R5 HIV-1_BaL_ strains, respectively. Technical cutoffs for the quantification of *env*-mediated cell fusion events were calculated as the mean plus two standard deviations (SD) of the β-galactosidase activity detected after HEK293T cells, transfected with 100% R5 HIV-1_BaL_ or 100%×4 HIV-1_NL4-3_, were incubated in cell-to-cell fusion experiments with U87.CD4.CXCR4 or U87.CD4.CCR5.cells, respectively.

### HIV-1 replicative fitness determination using viral growth kinetics analysis

The ability of the twelve p2-INT-recombinant viruses, plus the HIV-1_NL4-3_ wild-type control, to replicate in the absence of drug pressure was determined by measuring viral growth kinetics as described [49], [55]. Briefly, 3×10^6^ MT-4 cells were infected at a MOI of 0.01 IU/cell in one ml of culture medium, incubated for 2 hrs at 37°C in 5% CO_2_. HIV-infected cells were then washed two times with 1×PBS, then split to be cultured in triplicate wells of a 24-well plate (1×10^6^ cells/well). Culture supernatant was assayed for up to 30 days post-infection as described [56]. Viral replication was quantified using the slope of the growth curves and performing linear regression analysis derived from the equation *log(y) = mt+log(h*), where *y* is virus quantity (cpm), *t* is time in days, and *h* is the *y*-intercept (day 0). All slope values for each virus were used to calculate the mean, standard deviation, and 10^th^ & 90^th^ percentiles. Differences in the mean values were evaluated using a One Way Analysis of Variance test and the significance difference from the reference HIV-1_NL4-3_ virus calculated using the Bonferroni's Multiple Comparison Test (GraphPad Prism v.6.0b, GraphPad Software).

### Population (Sanger) sequence analysis

PCR products corresponding to the *gag*-p2/NCp7/p1/p6/*pol*-PR/RT/IN- and *env*-C2V3-coding regions of HIV-1 were purified with the QIAquick PCR Purification kit (Qiagen) and sequenced (population or global sequence) using AP Biotech DYEnamic ET Terminator cycle with Thermosequenase II (Davis Sequencing LCC, Davis, CA). Nucleotide sequences were analyzed using DNASTAR Lasergene Software Suite v.10.0.1 (Madison, WI).

### Deep sequencing of *gag*-p2/NCp7/p1/p6/*pol*-PR/RT/IN- and *env*-C2V3-coding regions

The three PCR products corresponding to the *gag*-p2/NCp7/p1/p6/*pol*-PR/RT/IN- (1,657 nt and 2,002 nt fragments) and *env*-C2V3- (480 nt fragment) coding regions of HIV-1 were sequenced using DEEPGEN, a novel HIV-1 genotyping and coreceptor tropism assay [43]. Briefly, the three amplicons were purified (Agencourt AMPure XP, Beckman Coulter) and quantified (2100 Bioanalyzer DNA 7500, Agilent Technologies) prior to using the Ion Xpress Fragment Library Kit (Life Technologies, Carlsbad CA) to construct a multiplexed library for shotgun sequencing on the Ion Personal Genome Machine (PGM, Life Technologies). For that, a mixture of all three purified DNA amplicons (33 ng each) was randomly fragmented and blunt-ends repaired using the Ion Shear Plus Reagent (Life Technologies) followed by DNA purification (Agencourt AMPure XP, Beckman Coulter). The P1 adapter and one of 12 barcodes were ligated to the repaired fragment ends prior to DNA purification (Agencourt AMPure XP, Beckman Coulter). DNA fragments were then selected by size (i.e., 280 to 320 bp; Pippin Prep, Life Technologies) and each barcoded library, i.e., a mixture of all three amplicons per sample, was purified (Agencourt AMPure XP, Beckman Coulter) and normalized using the Ion Library Equalizer Kit (Life Technologies). All thirteen barcoded DNA libraries, corresponding to twelve patient-derived amplicons plus the HIV-1_NL4-3_ control, were pooled in equimolar concentrations and templates prepared and enriched for sequencing on the Ion Sphere Particles (ISPs) using the Ion OneTouch 200 Template Kit v2 (Life Technologies) in the Ion OneTouch 2 System (Life Technologies). Templated ISPs were quantified (Qubit 2.0, Life Technologies) and loaded into an Ion 316 Chip (Life Technologies) to be sequenced on the Ion PGM using the Ion PGM Sequencing 200 Kit v2 (Life Technologies). Following a 4 hour and 20 minutes sequencing run, signal processing and base calling was performed with Torrent Analysis Suite version 3.4.2.

### Read mapping, variant calling, and phylogenetic analysis

Reads were mapped and aligned against sample-specific reference sequences constructed for the two genomic regions (*gag*-p2/NCp7/p1/p6/*pol*-PR/RT/IN- and *env*-C2V3) using the DEEPGEN Software Tool Suite as described[43]. The frequency of each amino acid present in each HIV-1 genomic position was calculated and summarized in a graphical interface with particular focus on sites of known drug resistance based on the latest edition of the IAS-USA HIV Drug Resistance Mutations list [57]. A list of the amino acids at these positions, and their frequencies, was exported as a tabulated text file and used with the HIVdb Program Genotypic Resistance Interpretation Algorithm from the Stanford University HIV Drug Resistance Database (http://hivdb.stanford.edu) to infer the levels of susceptibility to protease, reverse transcriptase, and integrase inhibitors. In addition, for each dataset, reads spanning amino acid positions (i) 50 to 85 in the protease (HXB2 2,400 to 2,508), (ii) 180 to 215 in the RT (HXB2 3,087 to 3,195), (iii) 130 to 165 in the integrase (HXB2 4,617 to 4,725), and (iv) 1 to 35 in the V3 region (HXB2 7,110 to 7,217) were extracted, truncated and translated for phylogenetic analysis and HIV-1 coreceptor tropism prediction as described below. Within each dataset only one representative of any identical variant was maintained, but the overall frequency stored. All variants with a frequency >1 within the population were aligned using ClustalW [58] and phylogeny reconstructed using the neighbor-joining statistical method as implemented within MEGA 5.05 [59]. In this study, minority variants were defined as amino acid substitutions detected at >1% (based on the intrinsic error rate of the system as described [43]) and <20% of the virus population, corresponding to those mutations that cannot be determined using population sequencing [25], [26], [27], [28], [29].

### Genotypic HIV-1 coreceptor tropism determination

As described for DEEPGEN [43], reads spanning amino acid positions 1 to 35 in the V3 region (HXB2 7,110 to 7,217) were extracted and truncated for HIV-1 coreceptor tropism determination using Geno2Pheno [60] with a FPR of 3.5% based on optimized cutoffs for determining HIV-1 coreceptor usage as previously described [33], [34], [61]. Deep sequencing V3 sequences usually spanned 105 nucleotides (35 amino acids), with some minor discrepancies associated with natural HIV-1 variation [62], [63], which led to V3 sequences with an open reading frame of 96, 99, 102, 108, or 111 nucleotides, all starting and ending with a cysteine codon, i.e., TG(T/C). V3 reads with stop codons (TGA, TAA, or TAG) and/or where the nucleotide length was not a multiple of 3 (e.g., 101, 103, 104, 106, etc.), mostly associated with natural or methodology (PCR or sequencing)-induced insertions and/or deletions, were not included in the analysis. Plasma samples were classified as containing non-R5 viruses if at least 2% of the individual sequences, as determined by deep sequencing, were predicted to be non-R5 [33], [34].

### Statistical Analyses

Descriptive results are expressed as median values, standard deviations, and confidence intervals. Pearson's correlation coefficient was used to determine the strength of association between categorical variables. All differences with a *P* value of <0.05 were considered statistically significant. As described above, differences in the mean of the slope values for the viral growth kinetics curves were determined using a One Way Analysis of Variance test and the significance difference from the reference HIV-1_NL4-3_ virus calculated using the Bonferroni's Multiple Comparison Test. All statistical analyses were performed using GraphPad Prism v.6.0b (GraphPad Software, La Jolla, CA) unless otherwise specified. *gag*-p2/NCp7/p1/p6/*pol*-PR/RT/IN and/or *env*-V3 nucleotide sequences obtained by deep sequencing in this study have been submitted to the Los Alamos National Laboratory HIV-DB Next Generation Sequence Archive (http://www.hiv.lanl.gov/content/sequence/HIV/NextGenArchive/Gibson2014).

## Results

### Antiretroviral drug susceptibility of multidrug-resistant viruses determined using HIV-1 genotyping (based on Sanger sequencing) and phenotyping assays

Plasma samples from 12 HIV-infected individuals experiencing virologic failure while participating in a 48-week dose-ranging study of elvitegravir were originally analyzed using standard (Sanger) population sequencing and an HIV-1 phenotyping assay (PhenoSense, Monogram Biosciences) [47], [48]. Here, we used patient-derived PCR products from the same plasma samples to construct *gag*-p2/NCp7/p1/p6/*pol*-PR/RT/IN-recombinant viruses and assess their susceptibility to 22 antiretroviral drugs, including EVG and RAL, using an alternative HIV-1 phenotyping assay (VIRALARTS) [49] (Table 1). A complete list of all amino acid substitutions in the protease, RT, and integrase coding regions, determined directly from plasma-derived amplicons using Sanger sequencing, is included in Table S1. Most of these highly treatment-experienced patients failed the antiretroviral regimen with viruses carrying multiple drug resistance mutations to protease (mean 4; range 0 to 10), nucleoside reverse transcriptase (3.8, 0 to 7), nonnucleoside reverse transcriptase (0.8, 0 to 3), and integrase (2.3, 1 to 4) inhibitors (Table 1). Most of the resistance mutations in PR and RT were present prior to initiating therapy with the EVG-based regimen (data not shown). The reduction in susceptibility to several antiretroviral drugs was corroborated by both HIV-1 phenotypic assays. For that, we compared the mutation scoring generated by the HIVdb Program Genotypic Resistance Interpretation Algorithm from the Stanford University HIV Drug Resistance Database (http://hivdb.stanford.edu) for each drug with the fold-changes in EC_50_ values (FC) obtained with the HIV-1 phenotyping assays. In general, there was good agreement between the Sanger sequencing-based genotype and the PhenoSense or VIRALARTS tests (*r* values of 0.89 or 0.87, respectively, *p*<0.0001 Pearson coefficient correlation). As expected, a strong statistically significant correlation was observed between the EC_50_ fold changes obtained with both HIV-1 phenotyping assays (*r* = 0.99, *p*<0.0001 Pearson coefficient correlation).

### Replicative fitness of multidrug-resistant *gag*-p2/NCp7/p1/p6/*pol*-PR/RT/IN-recombinant viruses

**Figure 1:**
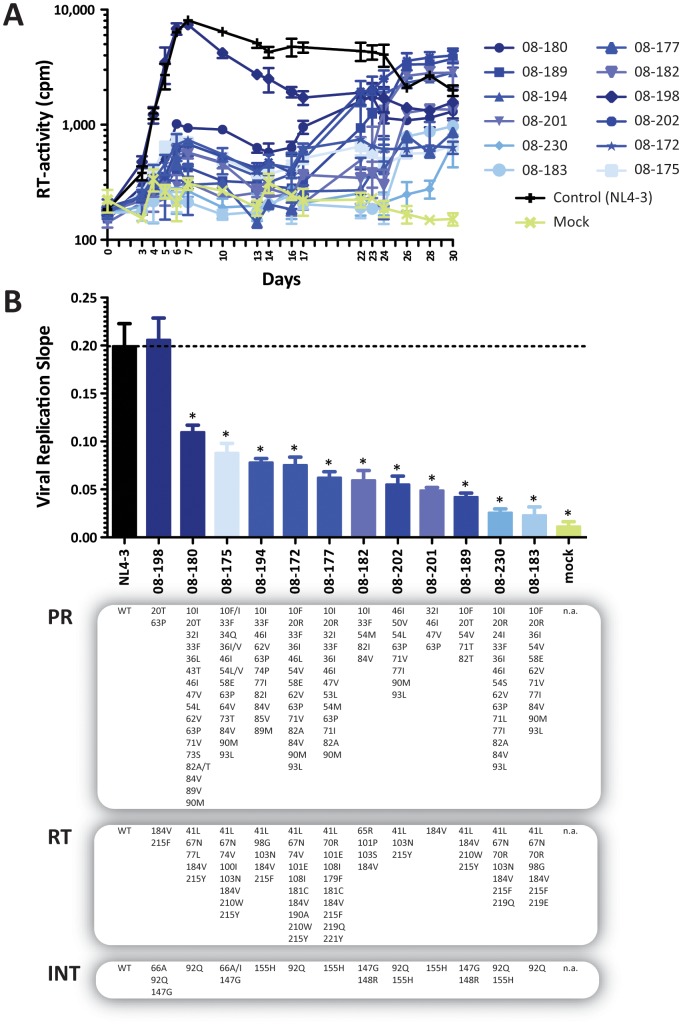
Replicative fitness of 12 patient-derived p2-INT-recombinant viruses in the absence of drug pressure. (A) Thirteen p2-INT-recombinant viruses (i.e., 12 patient-derived and the HIV-1_NL4-3_ wild-type virus) were evaluated for their ability to replicate in MT-4 cells in the absence of drug pressure. Virus replication was quantified by measuring reverse transcriptase (RT) activity in the cell-free supernatant. (B) Viral replication slopes were calculated using the slopes between RLU values at days 0 & 3, 0 & 4, 0 & 5, 0 & 6, and 0 & 7, corresponding to exponential viral growth. All five slope values for each virus were used to calculate the mean, standard deviation, and 10^th^ & 90^th^ percentiles. Differences in the mean values were calculated using a One Way Analysis of Variance test and the significance difference from HIV-1_NL4-3_ calculated using the Bonferroni's Multiple Comparison Test. The replication kinetics of viruses marked with an asterisk (*) were significantly different to the HIV-1_NL4-3_ control (p<0.05, 95% CI). Mutations in the protease (PR), reverse transcriptase (RT), and integrase (INT) coding regions are indicated for each virus. WT, wild-type (HIV-1_NL4-3_ virus).

All 12 replication-competent *gag*-p2/NCp7/p1/p6/*pol*-PR/RT/IN-recombinant viruses, generated for drug susceptibility analysis with VIRALARTS, were used in viral growth kinetic experiments to determine their replicative fitness in the absence of drug pressure. Statistical analysis of the slope of the growth curves showed that the replicative fitness of most multidrug-resistant viruses was statistically significantly reduced compared to the reference HIV-1_NL4-3_ strain (Fig. 1). The differences in replicative fitness among the recombinant viruses were patient-dependent and guided mainly by the number and type of drug resistance mutations in the PR, RT, and INT coding regions (Fig. 1B and Table S1). Interestingly, the replicative fitness of the 08-198 recombinant virus was not impaired relative to the wild type HIV-1_NL4-3_ strain, despite carrying primary drug-resistance mutations in the reverse transcriptase (M184V + T215F) and integrase (T66A + E92Q + S147G) coding regions (Fig. 1B). Nevertheless, there was a tendency –although not statistically significant– for viruses with higher mutation scoring determined by Sanger sequencing to have reduced viral replicative fitness values in the absence of drug pressure (*r* = −0.2, *p* = 0.16, Pearson coefficient correlation). Similar results were observed when viral replicative fitness values were compared with EC_50_ fold changes determined with either one of the HIV-1 phenotyping assays, i.e., PhenoSense or VIRALARTS (*r* values of −0.14, *p* = 0.23 or −0.13, *p* = 0.21, respectively, Pearson coefficient correlation).

### Determination of antiretroviral drug susceptibility using deep sequencing

**Figure 2:**
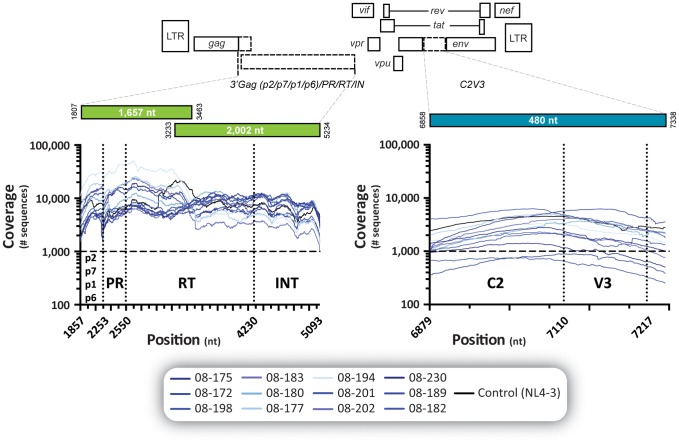
Coverage, i.e., number of reads per nucleotide position, obtained by deep sequencing the 12 patient-derived and the HIV-1_NL4-3_ wild-type virus. The *gag*-p2/NCp7/p1/p6/*pol*-PR/RT/IN and *env*-C2V3 fragments from all thirteen viruses were RT-PCR amplified and sequenced as described [43]. See Materials and Methods for details.

We have recently developed a novel HIV-1 genotyping and coreceptor assay based on deep sequencing (DEEPGEN) that allows the detection of minority HIV-1 variants when present at frequencies as low as 1% of the HIV-1 population [43]. Here we used this methodology to identify low-level drug-resistant viruses otherwise not detected by Sanger sequencing. All 12 samples were multiplexed into a single Ion 316 chip (59% loading efficiency of Ion Sphere Particles), generating 2,581,962 total quality reads and an average read length of 176 bp. Although comparable, the average sequencing coverage at each nucleotide position varied with each sample and HIV-1 genomic region analyzed, i.e., *gag*-p2/NCp7/p1/p6 (mean 8,940; range 3,881 to 11,647), protease (10,389; 5,319 to 13,299), reverse transcriptase (9,426; 4,389 to 18,055), integrase (6,562; 2,512 to 8,894), and *env*-V3 region (2,310; 1,026 to 3,270) (Fig. 2). These metrics ensured the minimum coverage of 1,000 per nucleotide position sequenced required to guarantee the detection of a minor variant present at least at 1% of the population [64].

**Figure 3:**
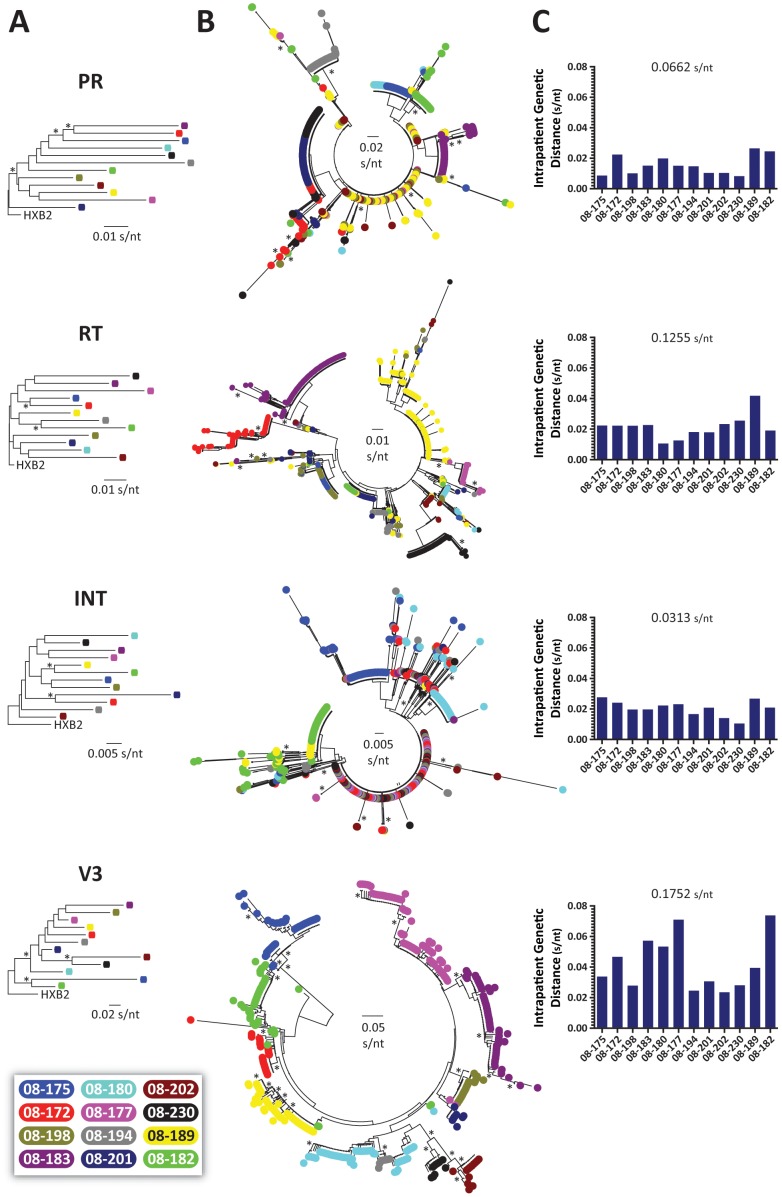
Phylogenetic and HIV-1 genetic diversity analysis. (A) Neighbor-joining phylogenetic trees constructed using population (Sanger) sequencing of 105-bp fragments corresponding to the HIV-1 protease, RT, integrase, and V3 regions from the 12 patients. Phylogenetic trees were rooted using the HIV-1_HXB2_ sequence (GenBank accession number AF033819). (B) Neighbor-joining phylogenetic trees constructed using reads with a frequency >1 corresponding to 105-bp fragments from the protease, RT, integrase, and V3 regions. Each color-coded dot represents a unique variant, frequency is not depicted. Bootstrap resampling (1,000 data sets) of the multiple alignments tested the statistical robustness of the trees, with percentage values above 75% indicated by an asterisk. s/nt, substitutions per nucleotide. (C) HIV-1 intra- and inter-patient genetic diversities were determined using MEGA 5.05 [59].

We first constructed neighbor-joining phylogenetic trees using 105 bp fragments from the protease, RT, integrase, or V3 HIV-1 regions -obtained by Sanger sequencing- to rule out any potential cross-contamination (Fig. 3A). Next, all deep sequencing reads with a frequency >1 corresponding to the same protease, RT, integrase, and V3 HIV-1 regions were used to construct neighbor-joining phylogenetic trees to quantify intra- and inter-patient genetic distances. A total of 2,482 unique protease (mean 207; range 80 to 581), 2,325 unique reverse transcriptase (194; 64 to 730), 1,462 unique integrase (122; 64 to 186), and 695 unique V3 (58; 22 to 75) sequences were included in each phylogenetic analysis. A clear virus-dependent clustering of unique sequences was evident only in the V3 region, while certain protease, RT, and integrase HIV-1 sequences from different patients branched together due to the multiple drug resistance mutations shared by the viruses obtained from these highly antiretroviral-experienced patients (Fig. 3B). Nevertheless, and as expected, interpatient genetic distances were larger than the range of intrapatient genetic diversity in the four HIV-1 regions, i.e., 0.0662 (0.0082 to 0.0264), 0.1255 (0.0106 to 0.0418), 0.0313 (0.0105 to 0.0276), and 0.1752 (0.0235 to 0.0738) substitutions per nucleotide in the protease, RT, integrase, and V3, respectively (Fig. 3C). Although not statistically significant, there was a tendency for viruses with higher genetic diversity in all four HIV-1 coding regions analyzed, i.e., more heterogeneous virus population, to have lower viral replicative fitness values (regression values ranging from -0.16 to -0.24, *p*>0.1, Pearson coefficient correlation). Interestingly, plasma viral load seems to be related to HIV-1 genetic diversity in the protease, RT, and integrase (*r* = 0.26, 0.26, and 0.18, respectively, *p*>0.1, Pearson coefficient correlation) but may correlate negatively with quasispecies heterogeneity as measured in the V3 region (*r* = −0.23, *p* = 0.19, Pearson coefficient correlation).

**Figure 4:**
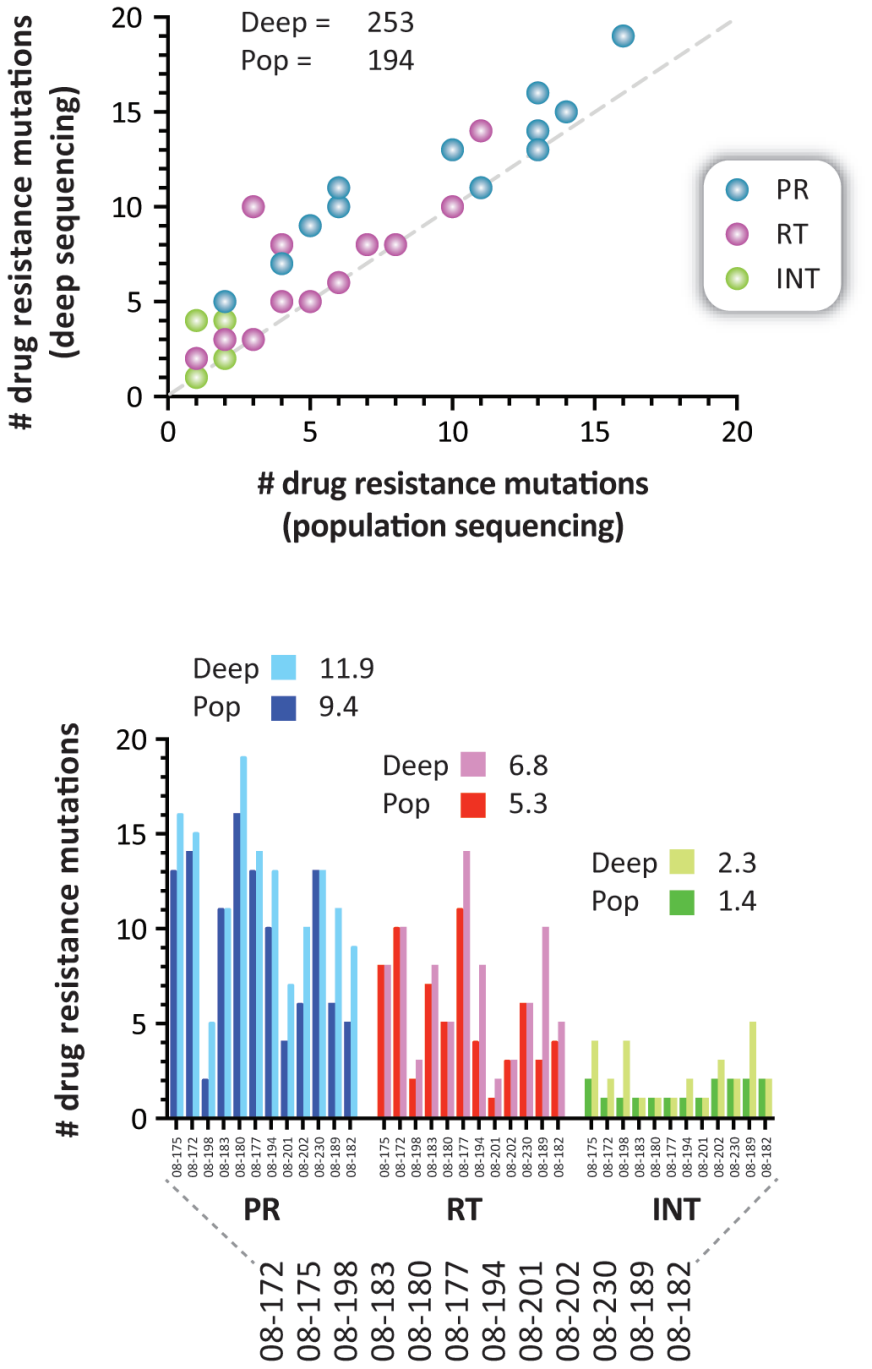
Comparison of the HIV-1 drug-resistant mutations identified by standard population (Sanger) and deep sequencing. Plasma samples from the 12 treatment-experienced HIV-infected individuals participating in the GS-US-183-0105 study of elvitegravir were analyzed as described in Materials and Methods. The top plot compares the number of drug resistance mutations detected by Sanger and deep sequencing in each patient. The total numbers of drug resistance mutations identified by each sequencing method are indicated. The mean difference in the numbers of drug resistance mutations detected by population and deep sequencing in the protease (PR), reverse transcriptase (RT), and integrase (INT) regions is indicated in the bottom graph. Deep, deep sequencing; Pop, population sequencing.

**Figure 5:**
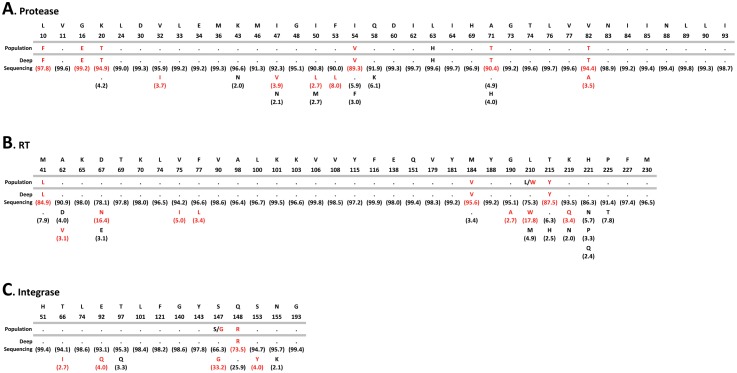
Frequency of amino acids detected in positions associated with HIV-1 drug resistance in the protease, reverse transcriptase, and integrase coding regions using Sanger (population) and deep sequencing (DEEPGEN) in patient 08–189. Amino acid substitutions associated with HIV-1 drug resistance are indicated in red.

Altogether a total of 194 mutations in positions associated with drug resistance were detected by both Sanger and deep sequencing, i.e., 113 in the protease, 64 in the RT, and 17 in the integrase (Fig. 4). As expected, all the drug resistance mutations identified by population sequencing were also detected by deep sequencing, while 59 additional drug resistance mutations were detected only by deep sequencing, i.e., 30 in the protease, 18 in the RT, and 11 in the integrase (Fig. 4 and Fig. S1). Overall, the difference in the numbers of drug resistance mutations detected by both methods was significant, even when the mutations were quantified by drug class, i.e., an average of 2.5, 1.5, and 0.9 additional mutations associated with PI, RTI, and INI, respectively, were detected by deep sequencing compared to population sequencing (Paired *t* test, *p*<0.0001) (Fig. 4). Figure 5 shows a comparison of the frequency of amino acids detected by population and deep sequencing in a virus carrying multiple mutations associated with resistance to PI, RTI, and INI (08–189). A number of amino acid substitutions associated with reduced susceptibility to antiretroviral drugs were identified only by DEEPGEN, all of them at frequencies below the limit of detection (<20%) of population sequencing [25], [26], [27], [28], [29], e.g., V82A (3.5%) in the protease, D67N (16.4%) in the RT, and E92Q (4%) in the integrase coding region. As exemplified in the integrase coding region, deep sequencing not only detected additional INSTI-resistance mutations in virus populations from 6 of the 12 HIV-infected individuals but was also able to quantify mixtures of amino acid substitutions identified qualitatively by population sequencing, e.g., E92Q (30.3%), N155H (59.9%) versus E92E/Q and N155N/H in patient 08–202, respectively (Table 2).

**Table 2:** INSTI-resistance mutations identified by population or deep sequencing in 12 HIV-infected individuals participating in the GS-US-183-0105 study of elvitegravir.

Patient	Population Sequencing a	Deep Sequencing (frequency [%]) a
08–175	T66T/A/I/V, S147S/G	T66A (71.1%), **L74M (3%)**, **E92Q (6.7%)**, S147G (74.6%)
08–172	E92E/Q	**T66A (4.4%)**, E92Q (55.1.%)
08–198	T66T/A, E92E/Q, S147S/G	**H51Y (11.6%)**, T66A (2.5%), E92Q (4.1%), S147G (90.5%)
08–183	E92Q	E92Q (99.8%)
08–180	E92Q	E92Q (96.7%)
08–177	N155N/H	N155H (79.9%)
08–194	N155H	**T97A (2.9%)**, N155H (99.8%)
08–201	N155H	N155H (98%)
08–202	E92E/Q, N155N/H	**T66I (3.8%)**, E92Q (30.3%), N155H (59.9%)
08–230	E92Q, N155H	E92Q (99.6%), N155H (98.5%)
08–189	S147S/G, Q148R	**T66I (2.7%)**, **E92Q (4%)**, S147G (33.2%), Q148R (73.5%), **S153Y (4%)**
08–182	S147G, Q148R	S147G (97.2%), Q148R (99.2%)

a Major mutations associated with resistance to INSTI as described [71], [86]. INSTI-resistance mutations identified using deep sequencing but not by population sequencing are indicated in bold.

As described above, we used the mutation scoring generated by the HIVdb Program Genotypic Resistance Interpretation Algorithm to compare the results obtained by deep sequencing with standard HIV-1 genotyping and phenotyping data, as well as clinical parameters. As expected, a strong statistically significant correlation was observed between the Sanger- and deep sequencing-based genotypes (*r* = 0.95, *p*<0.0001 Pearson coefficient correlation). Interestingly, the mutation scoring obtained by Sanger sequencing correlated slightly better with PhenoSense than with VIRALARTS (*r* = 0.89 and 0.78, respectively, *p*<0.0001 Pearson coefficient correlation), while a better agreement was observed between the mutation scoring calculated using deep sequencing and VIRALARTS than with deep sequencing and PhenoSense (*r* = 0.81 and 0.76, respectively, *p*<0.0001 Pearson coefficient correlation). Although not statistically significant, mutation scoring determined by deep sequencing tended to be higher in viruses with lower replicative fitness values (*r* = −0.23, *p* = 0.12 Pearson coefficient correlation). Finally, there was a statistically significant inverse correlation between HIV-1 drug resistance quantified by all four methods (Sanger sequencing, DEEPGEN, PhenoSense, and VIRALARTS) and plasma viral load, i.e., *r* = −0.70, −0.73, −0.62, and −0.65, respectively, *p*<0.0001 Pearson coefficient correlation.

### HIV-1 coreceptor tropism determination

**Figure 6:**
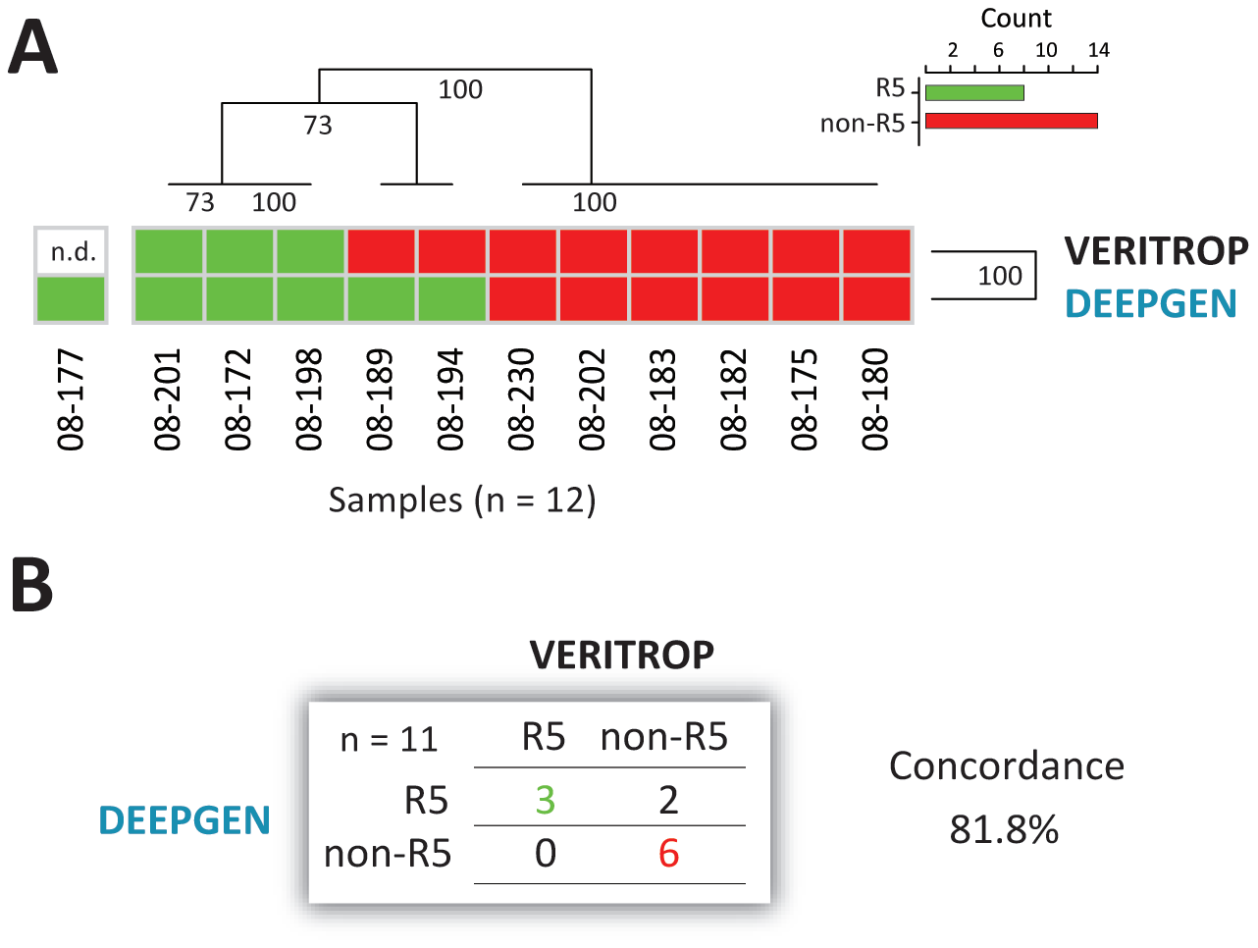
HIV-1 coreceptor tropism determination using deep sequencing (DEEPGEN [43]) and a phenotypic assay (VERITROP [54]). (A) Hierarchical clustering analysis was used to group the two HIV-1 coreceptor tropism determinations by similarity. Dendograms were calculated using the Euclidean distance and Complete cluster methods with 100 bootstrap iterations as described (http://www.hiv.lanl.gov/content/sequence/HEATMAP/heatmap.html). Bootstrap values are indicated. Green and red blocks indicate the absence or presence of non-R5 (X4) viruses, respectively, as determined by each assay. (B) Concordance between DEEPGEN and VERITROP.

In addition to identification of low-level drug resistance mutations, DEEPGEN is able to detect minority non-R5 (CXCR4-, dual-tropic, and/or dual mixed) HIV-1 variants [43]. Thus, here we used this deep sequencing-based assay to determine the coreceptor tropism of viruses obtained from the 12 HIV-infected individuals and compared the results with a phenotypic HIV-1 tropism test (VERITROP) [54]. Hierarchical clustering analysis grouped the two HIV-1 coreceptor tropism determinations based on their ability to detect R5 and non-R5 sequences, with 6 out of 12 patients harboring R5 HIV-1 strains based on deep sequencing analysis of the V3 region (Fig. 6A). In these patients, the concordance was high (81.8%) between the two HIV-1 tropism methods (Fig. 6B). Although not statistically significant (Paired *t* test *p* values ranging from 0.27 to 0.41), there was a tendency for non-R5 viruses to have a higher overall drug resistance level, based on the sum of the mutation scoring for all antiretroviral drugs (Sanger or deep sequencing) or EC_50_ fold-change values (PhenoSense OR VIRALARTS). Interestingly, the opposite was observed when HIV-1 coreceptor tropism was compared to plasma viral load, i.e., patients infected with R5 viruses seemed to have higher (while not significantly different), viral loads than patients carrying non-R5 viruses as majority members of the population (mean 359,150 copies/ml vs. 177,433 copies/ml, respectively; Paired *t* test, *p* = 0.101).

## Discussion

The clinical significance of minority HIV-1 drug-resistant variants is still under debate [30], [32], [39], [44], [45], [46], [65]. While a few studies have failed to establish an association between the presence of minority variants carrying drug resistance mutations and therapeutic failure [66], [67], [68], [69], many others have associated the identification of low-abundance drug-resistant viral variants as having an impact in treatment outcome [32], [35], [44], [45], [65], [70]. It is possible that the threshold for the identification of significant low-level variants may be mutation and/or antiretroviral drug dependent [31]; thus, it is clear that additional studies based on reliable ultrasensitive assays are needed to better understand the clinical relevance of these minority HIV-1 variants. Here we used a novel HIV-1 genotyping and coreceptor tropism assay based on deep sequencing [43] to quantify minority HIV-1 variants in patients experiencing virologic failure while participating in a 48-week dose-ranging study of elvitegravir and evaluated their contribution to HIV-1 replicative fitness and virological response to the antiretroviral treatment.

All 12 treatment-experienced HIV-infected individuals failed the INSTI-based therapy with viruses carrying a multitude of mutations conferring resistance to many PIs, NRTIs, NNRTI, and/or INSTIs. Among the primary INSTI-resistance mutations, standard population sequencing identified E92Q, N155H, and Q148R in 6, 5, and 2 patients, respectively. This is in agreement with previous studies showing that E92Q, Q148R/H/K, and N155H have been the most common EVG-resistance mutations identified *in vivo* [55], [71]. As expected, and similar to prior studies [49], [72], there was a good correlation between HIV-1 drug resistance determined by genotyping (Sanger sequencing) and phenotyping (PhenoSense or VIRALARTS) assays. Nonetheless, using a deep sequencing-based HIV-1 genotyping assay (DEEPGEN) [43] we were able to identify low-level HIV-1 variants (at a frequency of <20% of the population) carrying 59 additional drug-resistance mutations in the protease, reverse transcriptase and integrase coding regions of viruses from these highly antiretroviral-experienced patients. Previous studies have described similar findings, although analyzing no more than one or two drug-targeted regions at a time, in antiretroviral-naïve [65], [66], [73], [74], [75], [76] or -experienced HIV-infected individuals [43], [68], [69], [77], [78], [79], [80], with the detection of minority mutations usually correlating with historical antiretroviral treatment. In the case of INSTI-resistance mutations, using deep sequencing we corroborated and quantified all the amino acid substitutions identified by Sanger sequencing, even in positions labeled as mixtures, e.g., E92E/Q (55.1% E92Q) or N155N/H (79.9% N155H) in patients 08–172 and 08–177, respectively. More importantly, we identified 9 additional INSTI-resistance mutations at frequencies between 2.7% and 11.6% in 6 of the 12 patients, which most likely were or would have been playing a role in the overall susceptibility to INSTIs if the patients had continued in the antiretroviral regimen.

Are low-abundance HIV-1 drug-resistant variants contributing to virologic failure? As described above, additional minority drug-resistance mutations detected by deep sequencing have been shown to correlate with treatment failure [46], [70], [75], [76], [77]. Here we evaluated the relationship between the presence of drug-resistant variants, identified by Sanger or deep sequencing, and different virological and clinical parameters. First, the mutation scoring generated by the HIVdb Program using Sanger sequencing correlated slightly better with PhenoSense than with the other HIV-1 phenotyping test (VIRALARTS). On the other hand, a better agreement was observed between deep sequencing and VIRALARTS. VIRALARTS uses a yeast-based cloning methodology, which provides a better representation of the HIV-1 intra-patient population that other cloning methodologies [49], [81]. Thus, it is possible that additional minority HIV-1 variants, detected only by deep sequencing, were introduced during the yeast-based cloning step to generate the HIV-1 recombinant viruses. Second, although not statistically significant, viruses with higher drug-resistance score (determined by deep sequencing) had lower replicative fitness values, as it is usually the case for HIV-1 drug-resistant strains [21], [22], [24]. Interestingly, plasma viral load correlated negatively with the level of HIV-1 drug resistance determined not only by deep sequencing but also by the other three HIV-1 genotyping or phenotyping methods (*r* = −0.62 to −73, *p*<0.0001 Pearson coefficient correlation). The accumulation of primary drug-resistance mutations, although increases the replicative fitness of the virus in the presence of drug pressure, it does affect viral replication in the absence of antiretroviral drugs [21], [49], [82], [83]. Thus, although all patients experienced virologic failure with high plasma viral loads (mean, 268,291; range 35,400 tot 578,000 copies/ml), the addition of drug-resistance mutations seems to have a negative effect in overall viral replication. This finding was supported by the fact that plasma viral load correlated positively with HIV-1 genetic diversity in the protease, RT, and integrase (driven by the accumulation of drug resistance mutations) but it seems to correlate negatively with the *env*-V3 quasispecies heterogeneity, perhaps due to a bottleneck effect during the selection of HIV-1 drug-resistant variants [84], [85].

In summary, here we used a novel HIV-1 genotyping and coreceptor assay based on deep sequencing to analyze the contribution of minority HIV-1 drug-resistant variants in patients experiencing virologic failure while participating in a 48-week dose-ranging study of elvitegravir. We showed that these low-frequency drug-resistant viruses could enhance the overall burden of resistance not only to INSTI but also to PI, NRTI, and NNRTI. Should minority HIV-1 drug-resistant variants be considered when planning first-line or subsequent antiretroviral regimens? Detection of low-abundance NNRTI- or NRTI-resistant HIV-1 variants prior the initiation of antiretroviral therapy seems to correlate with a higher risk of virologic failure [32], [44], [45], [65], [70]. On the other hand, similar studies have not been able to associate the presence at baseline of low-frequency HIV-1 variants resistant to PI [67], NRTI [68], [69], or NNRTI [66] with antiretroviral therapy failure. To the best of our knowledge, no study has used deep sequencing to associate the effect of low-level INSTI-resistance variants prior the initiation of an INSTI-based antiretroviral regimen. Therefore, further and well-controlled longitudinal studies based on ultrasensitive HIV-1 genotyping assays are necessary to understand the clinical implications of minority HIV-1 drug resistance mutations.

## Supporting Information

Figure S1 **Amino acids detected in viruses from the 12 patients in codons associated with drug resistance in the protease, RT, and integrase regions according to the Stanford University HIV Drug Resistance Database (** http://hivdb.stanford.edu **).**
(DOCX)

Table S1 **Complete HIV-1 genotype for the 12 HIV-infected individuals participating in the GS-US-183-0105 study of elvitegravir.**
(DOCX)
