# Supplementary material for: Contribution of Human Immunodeficiency Virus Type 1 Minority Variants to Reduced Drug Susceptibility in Patients on an Integrase Strand Transfer Inhibitor-Based Therapy
Source: PLoS One. 2014 Aug 11;9(8):e104512. doi: 10.1371/journal.pone.0104512 (PMC4128663; doi:10.1371/journal.pone.0104512)
Supplement: Figure S1 — Amino acids detected in viruses from the 12 patients in codons associated with drug resistance in the protease, RT, and integrase regions according to the Stanford University HIV Drug Resistance Database ( http://hivdb.stanford.edu ). (DOCX) [file pone.0104512.s001.docx]

**Figure S1.** Amino acids detected in codons associated with drug resistance in the protease, RT, and integrase regions according to the Stanford University HIV Drug Resistance Database (<http://hivdb.stanford.edu>)


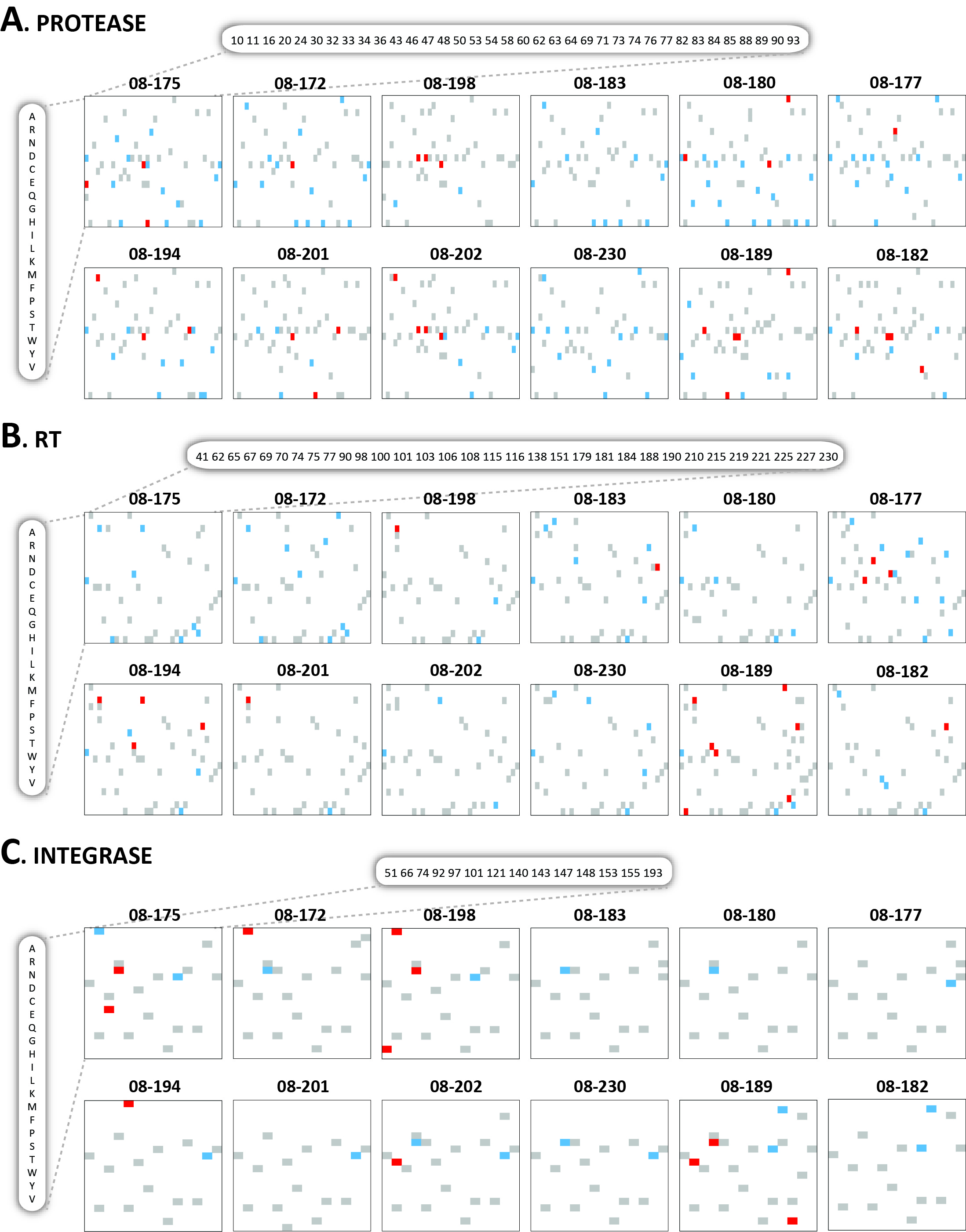


Drug resistance mutations with a frequency ≥20% (blue), >1% to <20% (red) or any other amino acid changes (grey) are indicated. Only amino acid substitutions with a frequency >1% are depicted.
