# Supplementary material for: Contribution of Human Immunodeficiency Virus Type 1 Minority Variants to Reduced Drug Susceptibility in Patients on an Integrase Strand Transfer Inhibitor-Based Therapy
Source: PLoS One. 2014 Aug 11;9(8):e104512. doi: 10.1371/journal.pone.0104512 (PMC4128663; doi:10.1371/journal.pone.0104512)
Supplement: Table S1 — Complete HIV-1 genotype for the 12 HIV-infected individuals participating in the GS-US-183-0105 study of elvitegravir. (DOCX) [file pone.0104512.s002.docx]

**Table S1.**  HIV-1 genotype for the 12 HIV-infected individuals participating in the GS-US-183-0105 study of elvitegravir.

| **Patient** | **Protease Sequence** | **RT Sequence** | **Integrase Sequence** |
| --- | --- | --- | --- |
| 08-175 | L10F/I K14R L33L/F E34Q E35E/D M36M/I/V M46M/I I54I/L/V Q58Q/E L63P I64V I66I/V I72I/M G73T I84V L90M I93L C95F/V | P9P/S V35M T39A E40F/S M41L K43E D67N L74V L100I Q102K K103N V118V/I K122E M184V T200T/A H208Y L210W R211K T215Y D218E K219N L228H V245E E248D A272P T286T/A A288S V293V/I E297K | S17N V31I T66T/A/I/V I73V L101I V113I T124N S147S/G I151V V234L A265A/V |
| 08-172 | T4S L10F L19L/I K20R L23I L33F E35D M36I R41K M46L I54V Q58E I62V L63P H69H/Y A71V I72V V82A I84V L90M Q92Q/K I93L | V35I/T M41L E44D D67N K70K/N L74V K101E Q102K V108V/I V118I K122E D123D/N C162D V179V/I Y181C M184V G190A E203K Q207D H208Y L210W R211K T215Y K219N K223E L228L/H A272P V292I | E11D D25E V31I L45L/P M50T V72I E92E/Q L101L/I V113I S119T I151V M154L H171H/L T206S S230N V234L |
| 08-198 | I13V I15V L19T K20T E35E/D L63P | K20R P25P/L K32K/R K49R I50I/V Q102K V118I I135T C162S T165I M184V T200A H208Y R211K F214L T215F A272P R277K R284K V293I E297K | S17S/N T66T/A L68L/I V72V/I E92E/Q L101I K111T V113I E138D S147S/G I151V K159K/E T206S E212A D232D/N V234L D253E Q274Q/R S283G |
| 08-183 | L10F T12S I15V L19V K20R E35D M36I I54V R57K Q58E Q61N I62V L63R K70E A71V V77I I84V L90M I93L | V35M/T M41L K43N V60I D67N T69D K70R A98G Q102K K104K/R V118I D123E C162S D177E M184V G196E T200A E203K E204E/D Q207E R211R/K F214L T215F/V K219E L228H V245M A272P K275Q V276I R277K | D3D/N E11D S17T A23V V72I E92Q T112V V113I I151V I203M Q216H V234L D256E |
| 08-180 | L10I I13V I15V K20T V32I L33F E35E/K M36L R41K K43T M46I I47V I54L I62V L63P I66F A71V G73S V82V/A/I/T I84V L89V L90M | K32K/E V35I M41L E44D S48T D67N V75M F77L A98S Q102K V118I K122P I135T C162S D177E M184V H208Y L210F T215Y K219N V261V/I A272A/P R277K A288A/T E297K | E11D K14R V31I L68L/V V72I E92Q L101I T112T/I V113L N117N/K I151V V201I T206T/S I208L V234L K240K/R D256E |
| 08-177 | P1P/H L10I I13V G16A L19L/V K20R V32I L33F M36I N37T M46I I47V F53L I54M K55R L63P A71I V82A L90M | E28K K32E M41L V60I K64R D67G T69N K70R L74I R83K K101E Q102K V108I P119P/R K122E D123N I135V E138Q T139T/I E169D I178L V179F Y181C M184V L187L/M K201K/R I202V T215F K219Q H221Y A272P R284K T286A E297K | V72I Q95Q/K V113I N117N/K S119S/R I151V N155N/H V234L D256E |
| 08-194 | L10I I13V L33F E35N N37E R41K M46I K55R R57K I62V L63P T74P V77V/I V82V/I I84V I85V L89M | K20R M41L S68S/G L74I V75T W88W/C A98G Q102K K103K/N V118I K122E C162S M184V R211K F214L T215F V245K V276I R277K L283I A288S V293I E297R A304E | E10D V72I P90A A91S L101I V113I S119R T124A E138E/K I151V N155H K156N V201I K211R V234L |
| 08-201 | I13V K14R V32I N37S R41K M46I I47V L63P I72V G86G/E | K11K/N/R/S V35V/I/M Q102K V118V/G D121Y K122E I135T K154K/R M164M/T P170P/T M184V L210F R211K A272P R277K P294A/S E297K A304E | K7Q E11D N27G V32I D41N V77V/A A91R L101V T112A V113I S119P T124N T125A I151V M154L N155H R166R/S T218S V234I |
| 08-202 | I15V P39P/L R41R/K M46M/I I50I/V I54I/L Q61E L63P A71V V77I L90M I93L | K20R M41L R83R/K Q102K K103N K122P I135T C162S D177E I178M G196E R211G T215Y K249K/E A272P R277K E297A | K14R L28I S39C E92E/Q L101I K103K/R V113I T124A I151V N155N/H I200M V201I V234L V249V/I D253E N254G |
| 08-230 | L10I I13V K20K/R L24I L33F M36M/I R41K M46M/I G48M I54S I62V L63P A71L I72V V77I V82A I84V I93L | E6E/K K20R M41L V60I D67N T69N K70R L74I K101S Q102K K103N K122E N136N/D C162S E169E/G D177E I178L M184V D192D/N G196E Q207H R211K T215F D218E K219Q A272P R277K P294Q E297S | S17N V72I E92Q L101I V113I F121F/S T124T/S I141I/V I151V N155H F181L K188R T206S I220L V234L N254N/S I268L R269K/R D270H |
| 08-189 | L10F K14K/R I15V G16E L19I K20T N37P I54V R57K L63H A71T V82T | P4S V35V/I T39A M41L R83K Q102K/R K122E C162S I178M M184V V189I H208H/Y L210L/W R211K T215Y A272P R277K Q278H E297R | V31I V54V/I A91A/V V113I E138E/K S147S/G Q148R I151V D167E K188K/R V201I T206S S230N V234L I251I/L A265V R269R/K |
| 08-182 | L10I I15V L33F I54M Q61H V82I I84V L89F | K65R S68G L74I R83K K101P Q102K K103S D123E C162S K173D/N Q174K I178L M184V D192D/N R211K V245E D250E S251D A272P V293I | G106A K111T V113I T124N E138K S147G Q148R I151V Y194C K211R N222K S230N V234L |

Patient-derived protease, RT, and integrase PCR products were originally sequenced using eight overlapping sequencing primers in an ABI37370 automated sequencer and compared to the reference HIV-1_NL4-3_ ([1](#_ENREF_1)). PCR products corresponding to the *gag*-p2/NCp7/p1/p6/*pol*-PR/RT/IN-coding region from the same patients were sequenced to confirm the original sequences using AP Biotech DYEnamic ET Terminator cycle with Thermosequenase II (Davis Sequencing LCC, Davis, CA). Nucleotide sequences were analyzed using DNASTAR Lasergene Software Suite v.7.1.0 (Madison, WI).

**REFERENCE**

1. **McColl DJ, Fransen S, Gupta S, Parkin N, Margot N, Chuck S, Cheng AK, Miller MD.** 2007. Resistance and cross resistance to fist generation integrase inhibitors: insights from a phase II study of elvitegravir (GS-9137). Antiviral Therapy **12:**S11.
